# Remote Assessment in healthcare—Technologies, methods, benefits, and challenges

**DOI:** 10.1371/journal.pone.0283945

**Published:** 2023-04-06

**Authors:** Jakob Eyvind Bardram

**Affiliations:** Department of Health Technology, Technical University of Denmark, Kongens Lyngby, Denmark; Dai Hoc Duy Tan, VIET NAM

## Abstract

The PLOS ONE Collection on “Remote Assessment” brings together a series of studies on how remote assessment methods and technologies can be used in health and behavioral sciences. At the time of writing (October 2022), this collection has accepted and published 10 papers, which address remote assessment in a wide range of health topics including mental health, cognitive assessment, blood sampling and diagnosis, dental health, COVID-19 infections, and prenatal diagnosis. The papers also cover a wide range of methodological approaches, technology platforms, and ways to utilize remote assessment. As such, this collection provides a broad view into the benefits and challenges of remote assessment, and *provides* a lot of detailed knowledge on how to make it work in practice This paper provides an overview of the included studies, and presents and discusses the different benefits as well as challenges associated with remote assessment.

## Introduction

Online and mobile research has grown exponentially over the last 30 years, providing easy access to wider and often hard-to-reach populations in an inexpensive, fast, and convenient way. This approach has gained particular relevance in handling the COVID-19 pandemic, in which the need to adapt experimental and clinical research to necessary physical distancing constraints has been paramount. These novel approaches for remote assessment in health and behavioral sciences have, however, also given rise to a set of new research challenges ranging from (re-)implementation of existing assessment forms, (re-)invention of novel assessment methods suited for interactive communication technology, establishment of reliability and ecological validity of these new assessment forms against golden standard methods, and the creation of stable, accurate, and secure technological solutions for remote assessment.

By the time of writing (October 2022), this PLOS ONE collection on “Remote Assessment” has accepted and published 10 papers which address a wide range of topics in remote assessment in topics related to health and behavioral sciences [1–10]. These papers covers a wide range of health domains, methodological approaches, technology platforms, and ways to utilize remote assessment. As such, this paper collection provides a broad view into benefits and challenges of remote assessment, and provide a lot of detailed knowledge on how to make it work in practice.

Not surprisingly given the timing and the wording of the call for papers, many of the papers address the COVID-19 lockdown and discuss how remote assessment methods can be used during a pandemic where people are more or less isolated and physical presence in clinics and between study participants needs to be minimized [1, 2, 4, 6, 7, 9]. As argued by Sacristán-Galisteo and colleagues [6], the COVID-19 pandemic has led to an unprecedented situation in which there has been a major push to implement telemedicine consultations, emphasizing the need for remote assessment tools, which offers the possibility to assess patients’ functional status without needing outpatient visits. As such, the lockdown has provides an unique opportunity for investigating remote assessment in many different health domain, applying many different methods.

This overview paper seeks to provide an overview of the 10 papers and summarize some of the findings. Please consult the individual papers for details and for important references to other related work in remote assessment methods and technologies.

## Overview—Health, technology, and methods

The papers in the collection address a wide spectrum of health topics including mental health [1, 10], cognitive assessment [5], blood sampling and diagnosis [9], dental health [8], COVID-19 infections [7], and prenatal diagnosis [3]. Two studies also address how remote assessment using online surveys can be implemented and used [2, 6], and one study review health inequalities in real-time remote outpatient consultations [4].

In the mental health domain, the study from Hong Kong applies a model-based approach to compare outcomes of guided internet-based cognitive behavioral therapy (i-CBT) against traditional face-to-face cognitive behavioral therapy (f-CBT) in a hypothetical cohort of university students with mild anxiety symptoms [1]. The model’s input parameters for clinical and utility were retrieved from published literature. The study finds that guided i-CBT appears to be cost-saving and effective for management of university students with mild symptoms of anxiety. Compared to traditional face-to-face CBT (f-CBT), offering guided i-CBT to manage students with mild anxiety symptoms reduced cost (by US $145 per case) and improved the quality of life for students, as measured by quality-adjusted life year (QALY) which gained in a five-year time horizon. The cost-saving in i-CBT is primarily due to lowered requirement of clinical psychologist staff-time and reduction in deteriorated cases. This is a promising result, since the mental health of university students has become a growing global concern. With the outbreak of the COVID-19 pandemic and the periodic lockdown measures to contain the spread of disease, psychological reactions to the pandemic have become prominent since.

The second mental health study is investigates if passively and remotely sensed data from smartphones can assess mental health symptoms from the context of patients’ lives [10]. Prior studies have shown that this is feasible. But these studies have trained models using data from single longitudinal studies, collected from demographically homogeneous populations, over short time periods, using a single data collection platform or mobile application. However, the generalizability of model performance across studies has not been assessed, i.e., if a model trained on one dataset can be used on another. This study demonstrates that machine learning models trained on combined longitudinal study data may generalize across heterogeneous datasets, which entails that passive and remote collection of data from smartphones can be used for prediction of mental health problems at a large scale.

The study in cognitive assessment investigates if such research can be transferred from the traditional laboratory settings to an online environment [5]. This study tests the validity of a web-based episodic memory paradigm by comparing participants’ memory performance in a supervised laboratory setting and an unsupervised web setting. Consistent with previous results, the study finds the memory performance to be comparable in the online and the laboratory setup, suggesting that web-based procedures for cognitive testing are a promising tool for memory research.

As for collection of survey data, there is a growing body of evidence that this can be done remotely. This is also demonstrated by the study by Sacristán-Galisteo and colleagues [6] who demonstrate that the web-based Spanish Post-COVID-19 Functional Status (PCFS) scale provided substantial test-retest reliability as compared to the paper-based version. The study by Sosenko and Bramley [2] similarly shows how methods for collection of survey data can be moved from a laboratory-based to a remote smartphone-based approach. The study demonstrates how the Respondent Driven Sampling (RDS) survey method can be implemented using smartphone technology while mitigating the possibilities for fraud, which comes when human verification in the clinic is absent in remote assessment.

From a technological point of view, remote assessment is often associated with the use of telephones, smartphones, video, or web-based technologies. This is also evident in this collections, where most studies use video/phone consultation [4], web-based systems [1, 5–7], or smartphones [2, 10]. Interestingly, however, a few of the included studies also involve remote assessment using medical devices.

In the study by Brandsma and colleagues [9], medical devices for self-administered blood micro-sampling were shipped to the participant at home for collection of blood samples. Self-collection devices have the potential to shift blood sampling closer to the point of care, supporting telemedicine strategies and virtual clinical trials. This study assesses a capillary blood micro-sampling device for measuring blood protein levels in healthy subjects and non-hospitalized COVID-19 patients. The study demonstrates the utility of remote serum collection to enable detection of elevated biomarkers of inflammation in the disease state of COVID-19 and they conclude that self-collection of capillary blood with micro-sampling devices provides an attractive alternative to routine in-clinic blood sampling. However, concentrations of certain analytes may differ significantly from those in venous samples, and factors including user proficiency, temperature control and time lags between specimen collection and processing need to be considered for their effect on sample quality and reproducibility.

The TestBoston study implements a fully remote, longitudinal, large-scale COVID-19 surveillance study [7] utilizing a nasal swap kit for PCR testing, a blood sampling device for collection of blood, and an online web-based portal for reporting exposure data. As such, this is an example of a multi-technology setup for remote assessment. More than 10,000 patients were enrolled in this study over a 4 month period. The study clearly demonstrates how a remotes assessment model like the TestBoston study enables fast, efficient enrollment and collection of longitudinal infectious disease surveillance data during the lock-down. This would not have been feasible using an in-person approach, which would have required over 44,000 discrete study visits.

Most of the the studies in the collection involve patients and citizens as the “remote” part of the remote assessment. The study by Beldjerd and colleagues [3] is, however, an exception. This is a more ‘classical’ telemedicine setup, where one clinician gets (remote) assistance from another clinician. In this study, a midwife sonographer is recording fetal ultrasound images and in case any abnormalities were detected, the images can be send to an expert physician for further analysis and diagnosis. An approach they call “asynchronous tele-expertise” (ASTE). The aim of the study is to assess the potential of the use of ASTE to provide prenatal diagnosis from a medical and economic point of view. The study finds that the use of asynchronous telemedicine in fetal ultrasound is feasible and may contribute to increased diagnostic accuracy. Moreover, and in contrast to other studies, this study also investigates the economical cost/benefit of this telemedicine setup, and shows that it generates a significant reduction in costs for society.

Finally, most of the included studies address remote assessment in a clinical setting involving patients and clinicians. However, a few of the papers address remote assessment in ‘virtual’ or ‘decentralized’ clinical trials—a topic which is gaining an increasing interest from a commercial point of view from the pharmaceutical companies and the clinical research organization (CRO) supporting them. The study by Brandsma and colleagues [9] shows how the deployment of self-collection devices for blood micro-sampling can shift disease diagnosis and population monitoring closer to the point-of-care, and such an approach has the potential to “revolutionize […] virtual clinical trials, providing conveniences to the patient and enabling outreach to remote populations” [p. 2]. Similarly, the authors of the TestBoston study [7] argue that remote assessment is key in running remote trials: “[T]he decentralization of clinical trials offers tremendous potential to disrupt the clinical trial landscape by reaching more representative cohorts and increasing scale, reducing per-participant time commitments for study staff, and promoting accessibility” [p. 11]. As such, remote trial models may reduce barriers to research engagement, improve retention, and reach a more representative cohort.

## Benefits of remote assessment

All of the included papers highlight a wide range of benefits to remote assessment. As summarized by Giraudier and colleagues [5]; remote assessment methods allows for the recruitment of large and diverse samples of participants in terms of age, gender, origin, culture and social status, minimizes organizational issues such as scheduling conflicts and time constraints, eliminates potential experimenter effects, and reduces costs related to travel, laboratory space, personnel hours, equipment, and administration.

### Recruitment and logistical benefits

A clear benefit to remote assessment is a better recruitment process. Remote assessment (i) allows the recruitment of large and diverse samples of participants in terms of age, gender, origin, culture and social status [2, 5], (ii) offers tremendous potential to engage representative cohorts, scale biomedical research, (iii) promotes accessibility by reducing barriers common in traditional trial design [7], and (iv) may help reach and drive uptake in under-represented participant groups [4] or groups that are “hard-to-reach” [2]. Moreover, some patient groups—like young university students—who are ‘digital natives’ may even prefer online platforms [1]. Remote trials and assessment methods also showed a higher retention rate [7].

From a logistical point-of-view, clinical trials faced unprecedented logistical barriers during the COVID-19 pandemic. These including social distancing protocols, restructuring of clinical sites to accommodate inpatient surges, participants’ fear of potential exposure during study visits, reduction of in-person research staff, and policies deeming study visits non-essential, necessitating adoption of remote methods to sustain research [7]. The studies in this collection show that rather being constrained by such limitations, remote assessment approaches help to minimizes organizational issues such as scheduling conflicts and time constraints, eliminates potential experimenter effects, and reduce costs related to laboratory space, personnel hours, equipment, and administration [5]. For example, in the TestBoston study [7]; “compared to in-person trials where participants travel to study sites and are guided through procedures, […] participants were able to independently navigate participation, including online registration, consent, survey completion, self-directed sample collection and shipment” [p. 6–7].

### Improved outcome

A number of studies also show equivalent or even improved outcome from the remote assessment approaches. In mental health, the Hong Kong study by You and colleagues [1] shows that compared to f-CBT, offering guided i-CBT to manage students with mild anxiety symptoms gained additional QALYs in a five-year time horizon. The QALY gain was generated by improved acceptance rate of CBT associated with i-CBT versus f-CBT and the consequently enhanced recovery. Similarly, the study by Beldjerd and colleagues [3] shows that remote telemedicine consultations in prenatal ultrasound diagnosis can be done in 91% of the included cases and they argue that if such remote assessment is put in place for more clinics, this may contribute to increased diagnostic accuracy.

### Economic benefits

A few of the studies also demonstrate economic benefits to remote assessment methods, including cost savings. The Hong Kong study [1] shows that the guided i-CBT method has cost-saving potentials for management of mild symptoms of anxiety. Compared to the f-CBT, offering guided i-CBT reduce cost by US $145 per case. This cost-saving is primarily due to lowered requirement of clinical psychologist staff-time and reduction in deteriorated cases, resulting in reduced utilization of outpatient and inpatient psychiatric care. Similarly, the ultrasound study by Beldjerd and colleagues [3] shows an average saving of 61.8% (€120) per patient compared to a face-to-face consultation, which is a “significant reduction in costs for society”. Figure 3 in the paper (p. 8) shows a breakdown of the cost saving and shows that the savings comes from two sources; reduction of face-to-face consultation time and reduction of transport. Hence, this saving is seen from a ‘societal’ perspective, which include the patient’s time and cost for transportation. However, seen from the hospitals point-of-view, the cost saving is merely related to the reduction in face-to-face consultation time.

### Other benefits

The studies in the collection also report on a set of other benefits to remote assessment methods. One example is better data collection and management in clinical trials since most of the remote assessment methodologies rely on digital technology. This will later ease and improve data analysis and ultimately help build (open?) datasets for research purposes. Another benefit mentioned in the ultrasound study [3], is the reduction of psychological stress for the patient. Remote asynchronous diagnosis can significantly contribute to reduce the psychological stress that could be induced by a false diagnosis by considerably reducing the time required to obtain an expert opinion. A third benefit also reported in the ultrasound study [3], is that remote assessment allows for training and education of clinical staff. The availability of remotely collected data which is stored for later use, potentially holds the option for using this data, including the annotations and diagnosis of the clinicians, for training of younger clinicians. Moreover, if the asynchronous setup used in the study is combined with synchronous video, then the midwife collecting the images will also be able to be ‘in the loop’ and hence learn from the experts.

## Challenges to remote assessment

The collection also includes studies which highlight a number of challenges to remote assessment. These challenges help us to understand what is important to consider when setting up remote assessment studies—like technical support—and also point to areas of improvements to the methodologies in use. And—maybe most importantly—these challenges also point to concerns about when to use remote assessment approaches and especially for which patient groups.

### Technology and tech support

Not surprisingly, several of the studies report on challenges related to the technological setup in remote assessment. In order to run the TestBoston trial [7], which enrolled 10,000 participants over a 4-month period, a quite sophisticated technological setup was used, including several sub-systems that need to be integrated. These include the Google Cloud platform, the Pepper system from the Broad Institute, REDCap, SendGrid, an authentication service, and other systems for handling the logistics of the test kits. Hence, the technological setup for such studies is quite demanding. A rather big team of software engineers and designers were involved in setting up and running this study.

Studies with less sophisticated technological requirements also report on technological challenges. For example, when running remote cognitive assessment, study validity may suffer from inherent limitations due to lack of experimental control and potential technical challenges (e.g., variations in internet speed and display settings) from the many different end-user devices (phones, tablets, laptops, desktops) and web-browsers [5].

Generalization across devices also poses a challenge. For example, the mobile sensing data analyzed in the study by Adler [10] reveal that there is a large difference in the two dataset, due to the fact that the data is collected from very different types of smartphones using different type of sensors, operating systems, and software. Hence, it is hard to generalize over such heterogeneous data and the authors conclude that combining heterogeneous data does not improves model performance compared to training a machine learning model on a larger homogeneous sample.

### Shifting the burden to the participant

An interesting observation in some of the studies is that when using remote assessment methods, some—or most—of the work in terms of recruitment, engagement, and retention is moved to the participant. This is most prominently discussed in the TestBoston study [7]: “While the remote and automated nature of the study design reduced many tasks that would have been performed by study staff in traditional in-person visits, the additional burdens experienced by participants led to higher than anticipated study staff support requirements” [p. 8]. Shifting the burden to the participant also proves to backslash. Over the relatively short study period (4 month), they received 11,500 emails from participant and had to employ eleven rotating support staff dedicated solely to answering hotline calls Monday through Friday during business hours, and had two MDs full time available for phone support. Despite that many studies claim that the use of web-based surveys is core to remote assessment, an interesting observation from the TestBoston study is that one of the most persistent issues was assisting participants in completing their monthly surveys. Hence, maybe such online forms and surveys are not so easy for participants to fill in, as we may think.

Two studies are applying test equipment and blood sampling devices, which are send to the participants [7, 9]. Clearly, in this case the burden of performing the nasal swap test and extracting blood samples is moved to the participants of the study. Moreover, also the logistical tasks of packing, shipping and tracking the samples are moved to the participants—tasks which are non-trivial. For example, in the US Military study [9], blood sampling test tubes are to be send with FedEx Priority Overnight in insulated containers with an ice pack—a non-trivial logistical task to comply to for a participant.

In some studies, even the recruitment of participants is shifted to the participants themselves. In the smartphone-based Respondent Driven Sampling (RDS) study [2], the recruitment burden is shifted to the participants by using the participants network for ‘snowballing’. The participants are now engaged in the recruitment process, and the study shows that this will only work if proper incentives (i.e., payment) is associated with it.

### Health inequality

A single, but rather interesting study, in the collection looks at health inequalities in invitation and uptake in real-time remote outpatient consultations in secondary and tertiary care in the UK [4]. Through a systematic review of 29 studies, this review reveals several findings. First, that most of the reviewed studies report an increase in the uptake of remote consultations over time, that younger patients were significantly more likely to use video consultations compared to older patients, and that women were more likely to use remote consultations than men. However, the review also indicates that remote consultations may still perpetuate or exacerbate existing health inequalities in access to and the uptake of healthcare for some patient populations. The review found that in general, patients who are older in age, men, have lower household incomes, are unemployed, have lower educational attainment, are from an ethnic minority group, live in a rural location or do not speak English as their first language are less likely to engage with remote consultations Hence, the authors raise a word of warning: “Offering remote consultations may perpetuate or exacerbate existing health inequalities in access to healthcare” [p. 2].

### Other challenges

The study by Sosenko and colleagues [2] also brings forth an interesting challenge to remote assessment, namely the notion of fraud. The problem is that when studies are done online, it is cheaper and faster but under a serious threat from fraud, compromising data quality and validity of findings. Especially, if there is an economical incentive structure in place in a study this will open up for fraud from participants. For example, if there is a monetary compensation for filling in a survey, the design needs to prevent the same person to register multiple times or filling in multiple copies of the same survey, in order to collect the fee multiple times. In a worst-case scenario, this could generalize to a brute-force attack, where a computer script automatically generates millions of user accounts and fill in millions of surveys. Hence, the technology for participant recruitment and data collection should be designed to maximizes fraud prevention while still benefiting from low cost and speedy data collection.

Another challenge mentioned by Beldjerd and colleagues [3] is the lack of official reimbursement codes for telemedicine and remote assessment. In many healthcare management organisations (HMOs), reimbursement for a diagnosis or a consultation is still tied to physical presence in the doctor’s clinic. Hence, remote assessment of e.g., medical ultrasound images would not be reimbursed. Until such reimbursement structures are in place on a HMO or national level, remote assessment and diagnosis will not be implemented in clinical practice.

## Conclusion

The COVID-19 lockdown gave rise to the use of remote assessment in many situations and we now see that many of these approaches and technologies are increasingly being implemented also in regular clinical practice. Moreover, the idea of replacing traditional in-clinic clinical trial with remote clinical trials, seems to bring about both increased outreach, more efficient logistical setup, and reduced cost.

This paper collection has presented a wide range of studies that demonstrate the breath of this approach and the many benefits remote assessment brings. However, the studies also highlight a set of challenges to these types of studies, including the need for a very sophisticated technology setup and support, the problems of shifting a large burden on to the participant, and the potential reinforcement of health inequality in such studies.

We hope that these studies with the presented benefits and challenges may serve as an inspiration for improving remote assessment in health and behavioral science.
